# Control over size, shape, and photonics of self-assembled organic nanocrystals

**DOI:** 10.3762/bjoc.17.5

**Published:** 2021-01-06

**Authors:** Chen Shahar, Yaron Tidhar, Yunmin Jung, Haim Weissman, Sidney R Cohen, Ronit Bitton, Iddo Pinkas, Gilad Haran, Boris Rybtchinski

**Affiliations:** 1 Department of Organic Chemistry, Weizmann Institute of Science, Rehovot 76100, Israel; 2 Department of Chemical Physics, Weizmann Institute of Science, Rehovot 76100, Israel; 3 Current address: Center for Cancer Immunotherapy, La Jolla Institute for Immunology, La Jolla, CA, U.S.A.; 4 Department of Chemical Research Support, Weizmann Institute of Science, Rehovot 76100, Israel; 5 Department of Chemical Engineering, Ben-Gurion University, Beer Sheva 84105, Israel; 6 Ilse Katz Institute for Nanoscale Science and Nanotechnology, Ben-Gurion University, Beer Sheva 84105, Israel

**Keywords:** aromatic amphiphiles, exciton diffusion, organic nanocrystals, perylene diimides, self-assembly

## Abstract

The facile fabrication of free-floating organic nanocrystals (ONCs) was achieved via the kinetically controlled self-assembly of simple perylene diimide building blocks in aqueous medium. The ONCs have a thin rectangular shape, with an aspect ratio that is controlled by the content of the organic cosolvent (THF). The nanocrystals were characterized in solution by cryogenic transmission electron microscopy (cryo-TEM) and small-angle X-ray scattering. The ONCs retain their structure upon drying, as was evidenced by TEM and atom force microscopy. Photophysical studies, including femtosecond transient absorption spectroscopy, revealed a distinct influence of the ONC morphology on their photonic properties (excitation energy transfer was observed only in the high-aspect ONCs). Convenient control over the structure and function of organic nanocrystals can enhance their utility in new and developed technologies.

## Introduction

Semiconductor and metal nanoparticles exhibit size- and morphology-dependent properties arising from confinement effects and strong interactions between neighboring atoms [1–3]. The correlation between nanoparticle size and the related electronic and optical properties has extensively been studied, leading to applications in novel technologies and devices [4–6]. The development of the reprecipitation method [7] allowed the facile fabrication of (often crystalline) organic nano- and microparticles based on polydiacetylene [8], pyrazoline [9], perylene [10], and other molecules. In several cases, size-dependent absorption was reported [11–13]. These crystals found use in optoelectronic materials [14–16], as markers for imaging applications [12–13], and demonstrated anticancer properties [17]. However, control over the size and shape in such systems is challenging [8–19].

Surface chemistry methodologies allow improved control over crystalline product formation; however, these methods are indirect and limited by the nature of the interface involved in the process. For example, well-defined two-dimensional nanocrystals were obtained by the vapor transport method, resulting in improved charge mobility [20], but no control over the crystal size and morphology was demonstrated. Using self-assembled monolayers as templates for the seeding and growth of molecular crystals may offer control over structure and polymorphism [21]. However, in this method, the crystal formation is limited by the monolayer surface so that it does not allow facile bulk fabrication and restricts control over the crystal morphology [22–23]. Crystalline nanobelts assembled from perylene and perylene diimide (PDI) derivatives were reported, but their size and shape could not be controlled [24–25]. Modification of the building blocks in such systems results in a certain degree of control [26–27], yet the PDI nanobelts do not remain free-floating in solution and normally are characterized as solid-state materials [28], limiting the processability of the nanocrystals, the control over the morphology, and insights into the assembly.

In general, gaining control over the crystal formation represents a long-standing challenge [29–32]. In this respect, understanding and controlling the crystallization process is key to fabricating organic nanocrystals with a predesigned morphology and properties [33–35]. We have reported on 2D crystalline self-assembled systems based on a hierarchical assembly mode promoted by hydrophobic and π–π interactions [36]. Yet, the size and shape of these systems could not be controlled beyond the 2D morphology.

We report herein on the aqueous self-assembly of organic nanocrystals with a tunable aspect ratio. These systems are quite uniform and exhibit morphology-dependent photonics: strikingly, divergent exciton diffusion properties as a function of the shape.

## Results and Discussion

Following our interest in the self-assembly of PDI derivatives, we employed compound **1**, a PDI system with a hydrophilic group (phenoxybenzoic acid) attached to the aromatic core of PDI at the “bay area” [35]. Compound **1** is an asymmetric amphiphile that was designed to result in arrays that differ from fibrous and monolayer structures assembled from symmetrically substituted PDI systems [36–38]. Additionally, the aqueous self-assembly incorporating carboxylic acid groups in the covalent unit design has been shown to result in complex and tunable self-assembly modes [39–42].

### Crystalline self-assembly

We have found that the nonclassical crystallization of **1** in neutral aqueous solutions can be manipulated to result in different polymorphs [35], 3D crystals with dissimilar structures and morphologies. We envisaged that the crystallization of **1** in a basic aqueous medium can lead to 2D arrays (bilayers) due to the higher solubility of the assemblies as a result of the charged carboxylate groups that are expected to favor the solvation by water. We induced the self-assembly process by injecting a concentrated solution of **1** in THF (2 × 10^−3^ M) into basic water (pH 10) or a water/THF mixture to reach a 1 × 10^−4^ M concentration. We studied the following three assembly conditions: “10% THF”, i.e., injection of the stock solution to a basic-water/THF mixture to obtain a 10% THF content by volume; “5% THF”, i.e., injection of the stock solution to basic water to give a 5% THF content; “5%→0% THF”, i.e., injection of the stock solution to basic water to result in a 5% THF content, followed by the immediate evaporation of THF in a high-vacuum and adding water to reach a 1 × 10^−4^ M concentration.

**Figure 1:**
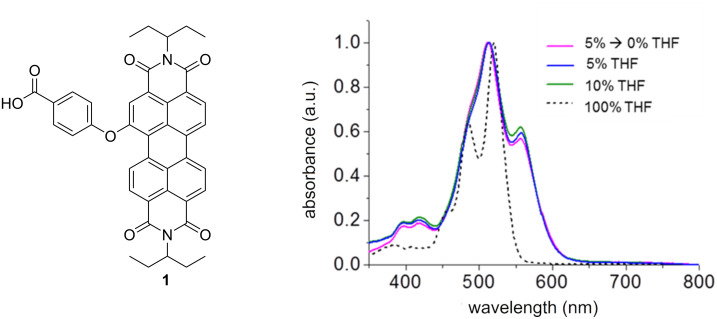
Chemical structure of compound **1** and UV–vis spectra in an aggregating aqueous medium and in the disaggregating solvent THF.

The self-assembly is instantaneous, as indicated by a color change from bright orange to pink in all systems. UV–vis spectra of compound **1** in aqueous medium exhibit a 0-0/0-1 vibronic band inversion, red shift, and significant broadening in comparison to the molecularly dissolved system (Figure 1B). This is a typical spectral signature of ordered PDI systems and crystals [38,43–47] having a face-to-face orientation of the π-systems.

**Figure 2:**
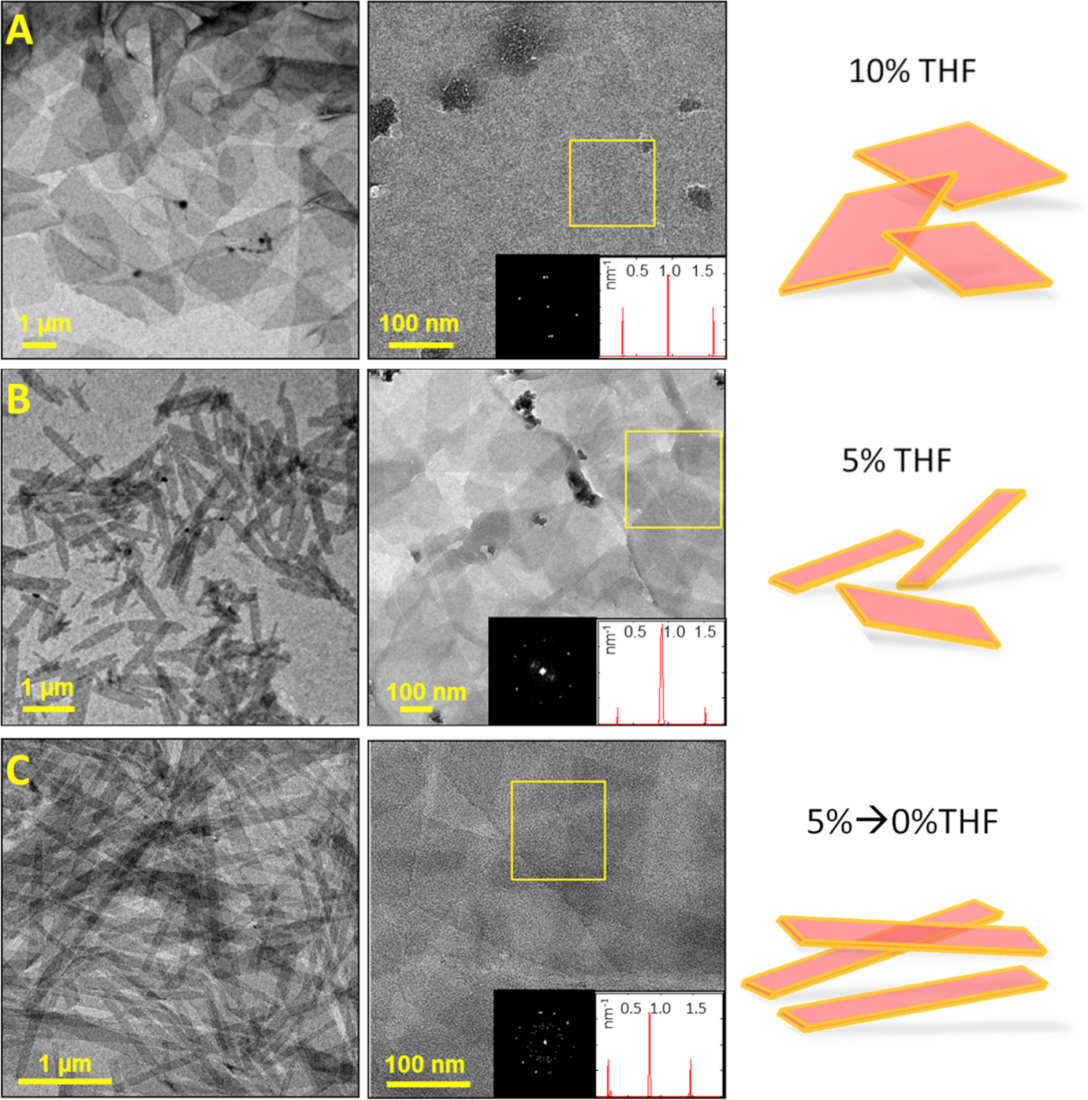
Transmission electron microscopy (TEM) images (left, zoomed-out and zoomed-in; 1 × 10^−4^ M solutions of **1** deposited on TEM grids) and crystal morphology sketches (right). A) 10% THF. B) 5% THF. C) 5%→0%THF. Insets: fast Fourier transform (FFT) analysis of the crystalline material (in the marked areas), demonstrating an identical spacing of 1.6 nm in all systems.

Cryogenic transmission electron microscopy (cryo-TEM) and TEM studies revealed that the 10% THF assemblies were long rectangular-shaped crystals, ≈3 µm in length (Figure 2A). The crystals had an aspect ratio of 2.6 ± 1.3, and their crystalline order was evident from FFT analysis, exhibiting well-defined spots corresponding to a periodicity of 1.6 nm. The 5% THF assembly gave rise to crystals that were ≈1 µm in length, with an aspect ratio of 5.0 ± 1.9. FFT analysis revealed the spacing value to be 1.6 nm, identical to the 10% THF system (Figure 2B). For the system 5%→0% THF, the crystals were over 5 µm long and under 0.5 µm in width, and thus having the largest aspect ratio amongst the studied systems, 10 ± 3.5. The crystalline order gave rise to a 1.6 nm spacing, as indicated by FFT (Figure 2C). The aspect ratio values and the calculated standard deviations are based on two different assembly solutions including 50 crystals for each system.

**Figure 3:**
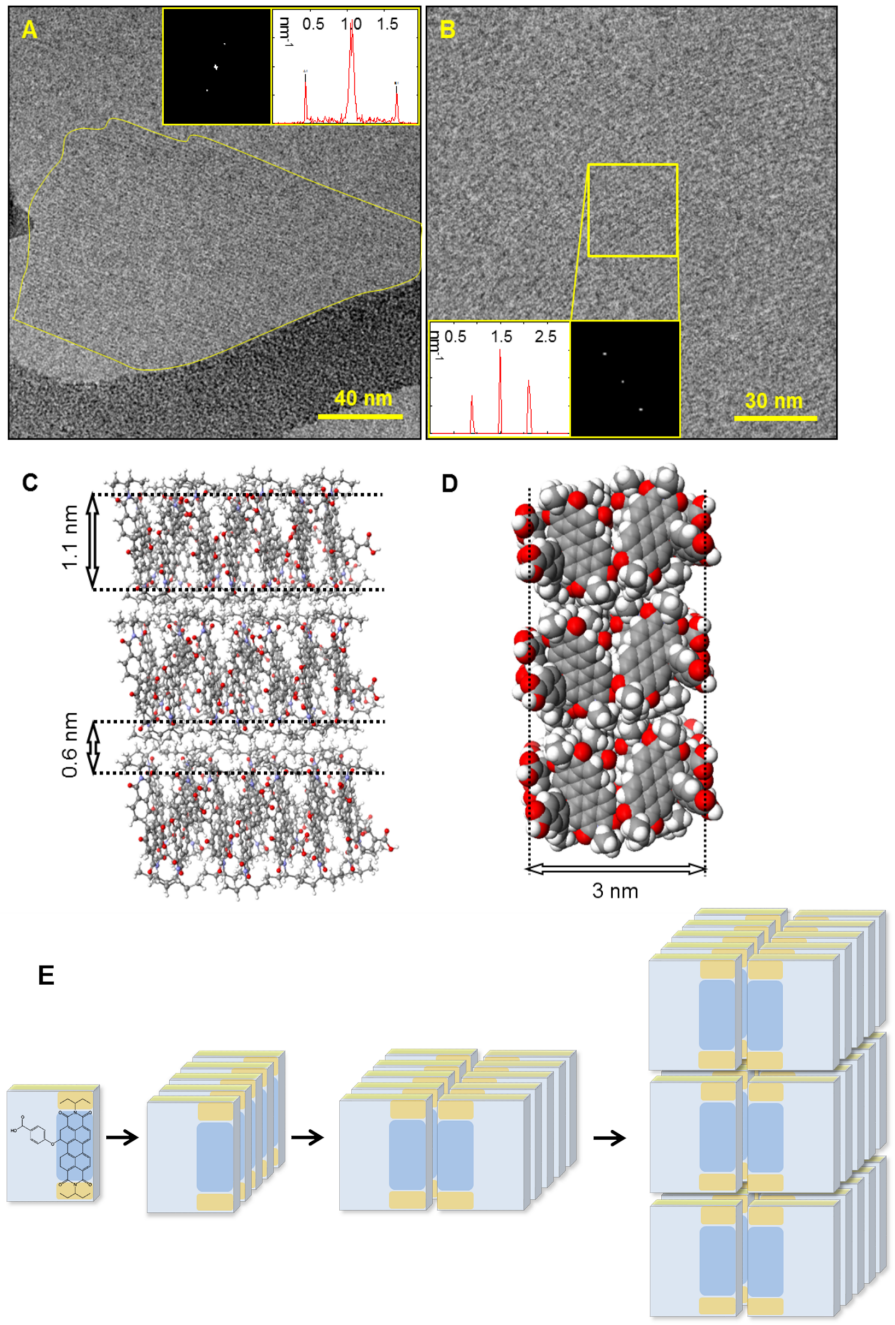
Cryo-TEM images of a 1 × 10^−4^ M solution of **1** (5% THF) and the corresponding molecular model as well as a cartoon illustrating of the self-assembly process. A) Cryo-TEM image, the crystal is highlighted with a yellow contour. B) Zoom-in showing the fine-structure composed of alternating dark- and light-contrast stripes. Insets: FFT analysis of the crystalline material, giving rise to a spacing value of 1.6 nm. C) and D) Molecular model: the interacting stacks and their dimensions corresponding to the observed fine-structure (C) and a cross-section demonstrating the bilayer structure composed of two adjacent PDI cores (D). The measured distance of 3 nm corresponds to the oxygen–oxygen distance between the carboxylic groups of two PDIs. E) Schematic representation of the assembly structuring.

All obtained crystalline assemblies demonstrated structural stability toward drying, and presented an identical fine structure composed of an ordered array of alternating dark and light-contrast stripes, as observed by TEM and cryo-TEM (Figure 2 and Figure 3, respectively). FFT indicates crystallinity (well-defined spots) and identical spacings in all systems. Image analysis revealed 0.96 ± 0.10 nm dark-contrast stripes separated by 0.69 ± 0.12 nm light-contrast stripes in all cases. The molecular model that best fits the cryo-TEM and TEM data is a bilayer, where pairs of PDI cores are coupled together, and the carboxylic groups of both PDIs turn outwards to the surrounding aqueous medium. The resulting 1D π-stacked bilayer structures interact via the ethylpropyl residues to form an extended bilayered crystalline sheet (Figure 3C,D,E). According to the model (Figure 3C,D,E), dark-contrast stripes correspond to the overlapping aromatic cores (1.1 nm), and the light-contrast stripes correspond to the ethylpropyl residues (0.6 nm), as observed in the cryo-TEM images (Figure 3A and Figure 3B).

In order to validate the bilayer width of the nanocrystals, atomic force microscopy (AFM) and SAXS measurements of the assembled compound **1** were employed. An air-dried sample of compound **1** deposited on a Si substrate (see Supporting Information File 1) gave rise to rectangular structures with well-defined edges (Figure S3, Supporting Information File 1), 3.2 ± 0.4 nm in height. This is in good agreement with the bilayer width in the molecular model, corresponding to the oxygen–oxygen distance between the carboxylic groups of two adjacent PDIs (Figures S2B and S2C, Supporting Information File 1). SAXS (of a 6 × 10^−4^ M aqueous solution of **1**, 5% THF, Figure S4, Supporting Information File 1) demonstrated a power law dependence of q^−2^ in the low q region, indicative of flat particles [48]. The background subtracted curve could be fit to a dilute lamellar form factor (Equation S3, Supporting Information File 1) to give an overall thickness of 35 Å ± 3 Å, which is in excellent agreement with the molecular modeling and the AFM results.

The presented crystalline structures are kinetically trapped products since they do not equilibrate upon changing the assembly conditions after they are fully formed. Thus, the addition of THF to preassembled 5%→0% THF crystals up to a 5% THF content did not result in any observable morphological change (Figure S6B, Supporting Information File 1). Similarly, when THF was added to the assembled 5% THF material to result in a 10% THF content, no change was observed (Figure S6A, Supporting Information File 1). Evidently, the structure of the crystals is defined by the assembly pathway rather than the equilibration at a given solvent composition. Apparently, hydrophobic interactions dominate the self-assembly, mitigating the repulsion of the charged carboxylate groups (partial protonation of the carboxylate moieties can also not be ruled out as a result of the hydrophobic self-assembly). We noted that at a lower pH (Figure S8, Supporting Information File 1), the self-assembly leads to different structures, probably due to the lower solubility and interactions between the protonated carboxylic groups. Crystals assembled under different pH conditions (Figure S8, Supporting Information File 1) are less homogeneous and demonstrate a larger size distribution than the crystals obtained by exerting control through the THF content.

**Figure 4:**
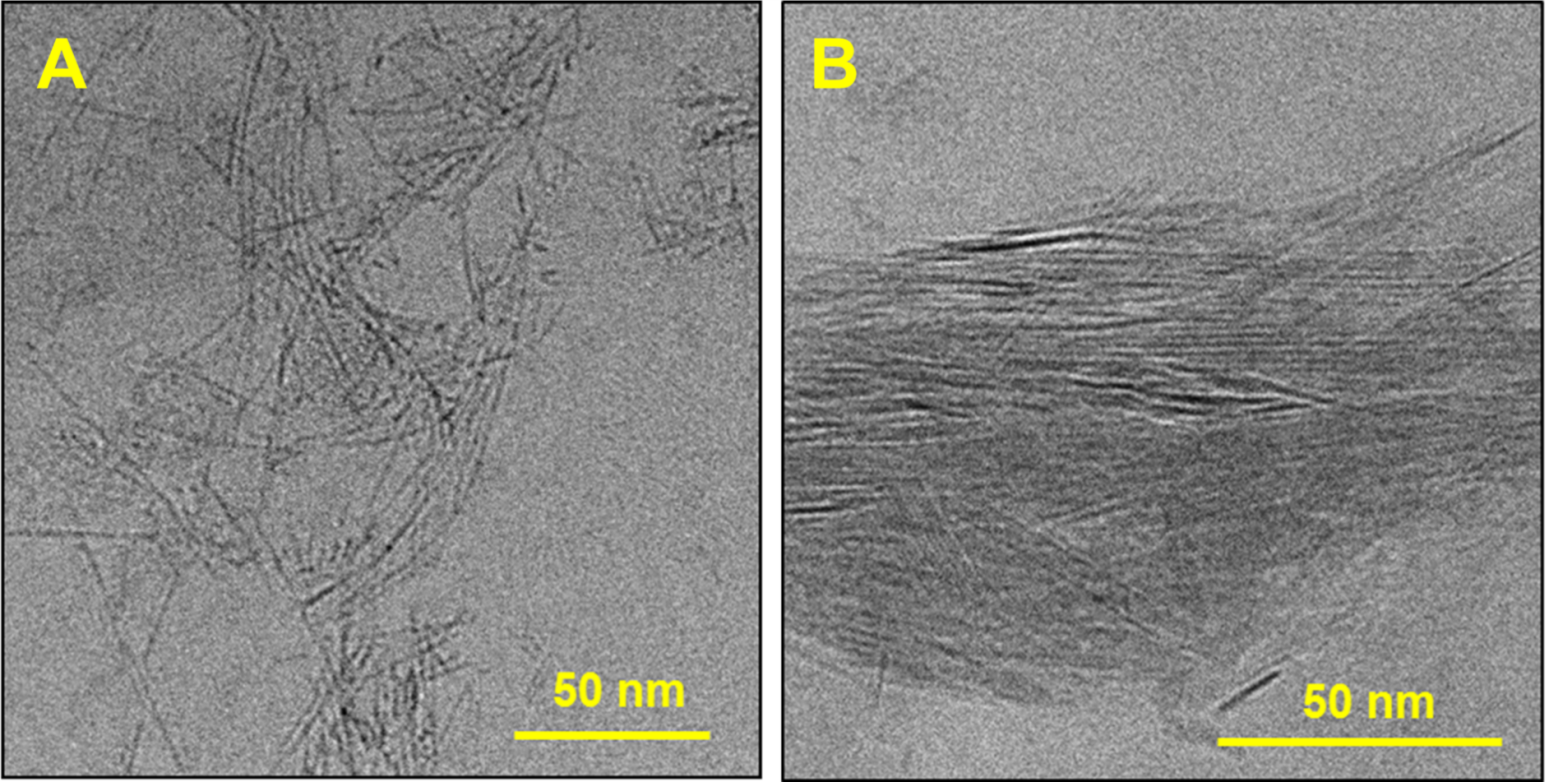
Cryo-TEM images of 1 × 10^−4^ M compound **1** in THF/water solutions after one minute of aging. A) 5% THF: monomolecular 1D π-stack fibers and B) 10% THF: crystalline platelets.

In order to gain insight into the crystallization mechanism and the effect of the THF concentration on the crystallization process, we performed cryo-TEM imaging of the early assembly stages. For the 5% THF assembly, monomolecular 1D stacks 1.5 ± 0.3 nm in width were observed after 1 min of aging (Figure 4A). The assembled material appeared as short fibers (10–80 nm length), some of which interact and align. For the 10% THF system, crystalline arrays ≈100 nm in length and ≈10 nm in width were observed after 1 min (Figure 4B). The structural differences between the 5% THF and 10% THF systems at early assembly times reveal the distinct dynamics of the nucleation and growth process. Thus, the 10% THF system shows a faster ordering process (THF renders the system more dynamic, lowering the activation barrier for a molecular reorganization [38,49]), resulting in long rigid fibers that interact (Figure 3E), leading to larger ordered arrays and templating the further assembly process. This, together with the better stabilization of the aromatic cores at a higher THF content, leads to large, low-aspect-ratio crystals in the 10% THF system. Thus, kinetically controlled pathway-dependent nucleation and growth defines the outcome of the crystalline self-assembly process. In both the 5% THF and 10% THF systems, the crystallization is largely completed after 5 min (Figure S7, Supporting Information File 1). The observed crystallization pathways are consistent with a gradual order evolution mechanism recently observed by us [34].

### Exciton dynamics

**Figure 5:**
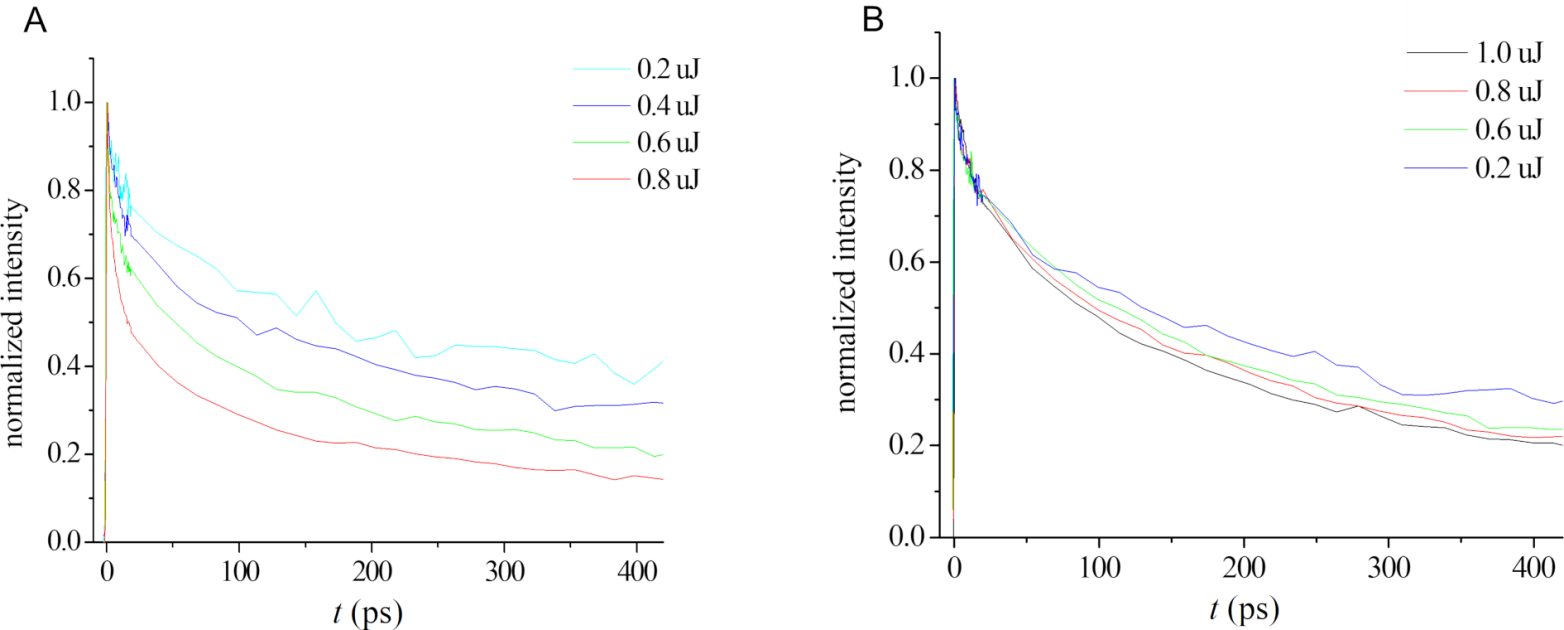
Transient kinetics at different laser powers probed at 755 nm (1 × 10^−4^ M solution at pH 10): A) 5% THF solution and B) 10% THF solution.

In order to investigate whether the morphology affects the photonic properties, we studied the excited state dynamics of the nanocrystals. Femtosecond transient absorption spectra of all assemblies displayed typical PDI excited state absorption peaks in the range of 600–770 nm [50], matching PDI bleaching represented by negative features at 550–600 nm. In the disaggregated state, almost no power dependence was observed. The decay kinetics of the 5% THF and 5%→0% THF systems demonstrated power dependence (Figure 5A and Figure S9A, Supporting Information File 1, respectively). In contrast, 10% THF nanocrystals exhibit essentially power-independent kinetics (Figure 5B). Power dependence is indicative of the exciton–exciton annihilation process, occurring when a high photon flux results in multiple excitons, which can efficiently diffuse through the aggregated material, resulting in exciton annihilation [51].

In order to validate the observed behavior and prove that the measured differences between the 5% THF and 10% THF assemblies stem from the different crystal morphologies and not from the differences in the THF concentration, a control experiment was performed. A sample of **1** prepared in 5% THF was aged for two days, after which the THF concentration was adjusted to 10%. The control sample was measured, showing exciton dynamics very similar to the original 5% THF sample (Table 1 and Figure S9B, Supporting Information File 1).

**Table 1:** Diffusion coefficient and exciton diffusion length.

measured crystal system	*D*, cm^2^/s	*L*_D_, nm^a^

5%→0% THF	0.07 ± 0.01	198 ± 4
5% THF	0.05 ± 0.01	170 ± 13
pre-aged 5% THF adjusted to 10% THF	0.04 ± 0.01	114 ± 14
10% THF	no power dependence	

^a^The exciton diffusion length is calculated using *L*_D_ = *D*⋅τ, where τ is the exciton lifetime (estimated as a longer decay component of the decay fit, representing a lower limit for the exciton lifetime).

In order to estimate the exciton diffusion coefficient and the diffusion length, we used an analysis method employed in our previous work [36] and based on a 1D diffusion model for the annihilation rate [52–55]. The model fits our data, revealing that exciton diffusion occurs primarily within 1D aromatic stacks, in agreement with the reported annihilation studies [54–55]. The fitted data is presented in Figures S10–S12, Supporting Information File 1, and the exciton diffusion parameters are given in Table 1.

The 5% THF and 5%→0% THF systems gave rise to similar diffusion coefficients: 5 × 10^−2^ cm^2^/s and 7 × 10^−2^ cm^2^/s, respectively. These values are comparable to the reported exciton diffusion coefficients of 2D PDI crystalline arrays reported by us [36] and those of perylenetetracarboxylic dianhydride (PTCDA) solid crystalline films (4 × 10^−2^ cm^2^/s) [41]. The exciton diffusion lengths in the assembled material are also comparable or higher than the reported values for 2D PDI crystals (120 nm) [36], PTCDA films (61 nm) [52], and PDI J-aggregates (96 nm) [56–57], indicating efficient exciton hopping. The 10% THF assembly showed almost no power dependence, in striking difference to other systems. This can be attributed to the exciton trapping [24], probably at the large aromatic “edges” in the 10% THF crystals.

Superresolution fluorescence microscopy measurements were carried out in order to further study the photonic behavior of the systems. Interestingly, the 5% THF system displayed an emission that was localized at regions that appeared to be matching the shape and dimensions of the crystal edges (Figure S13, Supporting Information File 1), with a clear preference for emission at the crystal corners (the microscopy of the 10% THF system did not provide reliable superresolution images). This further underscores the influence of the crystal morphology on the photonic properties [58] and will be a subject of future studies.

## Conclusion

We have demonstrated that a simple building block assembles into well-defined free-floating crystals in an aqueous medium. The crystalline assemblies are stable and their morphology can be fine-tuned as a function of the initial THF concentration or the pH in the assembly solution. The nanocrystals demonstrate morphologically-dependent photonic properties, showing uniquely dissimilar exciton diffusion behavior. Facile fabrication of well-defined nanocrystals combined with the ability to control their morphology and photonic properties represents a convenient structure/function tool advancing the applicability of organic nanocrystals.

## Experimental

### General information

The ^1^H and ^13^C NMR spectra were recorded at 20 °C on a 300 MHz NMR spectrometer (Bruker). Electrospray ionization (ESI) mass spectrometry was performed using a Micromass Platform instrument. UV–vis absorption and fluorescence measurements were carried out on a Cary-5000 spectrometer (Varian) and a Cary Eclipse fluorimeter (Varian), respectively.

### Preparation of the assemblies

Samples of compound **1** were prepared by dissolving the dry material in THF, followed by the injection of the THF solution into double-distilled water (Barnstead NANOpure Diamond water system, used for all sample preparations) at the desired pH (adjusted by adding a NaOH solution). The 5%→0% THF samples were prepared by an immediate evaporation of the THF cosolvent, followed by dilution with water to the desired final concentration. Clear homogeneous pink solutions were obtained.

### TEM

TEM imaging was performed using a Tecnai T12 transmission electron microscope operated at 120 kV.

**Sample preparation:** 8 µL of each sample was applied to a 400 mesh copper grid coated with carbon (on nitrocellulose support) and blotted 2 min later from the back side of the grid.

### Cryo-TEM

Cryo-TEM imaging was performed by a methodology analogous as described previously [36], using a Tecnai F20 transmission electron microscope operating at 200 kV and using a Gatan 626 cooling holder and a transfer station with a Gatan US4000 CCD digital camera or a Tecnai T12 transmission electron microscope operated at 120 kV, using a Gatan 626 cooling holder and transfer station, with a TVIPS F244HD CCD digital camera.

**Sample preparation:** 8 μL of each sample was applied to a 300 mesh copper grid coated with holey carbon (Pacific Grid-Tech supplies). The samples were blotted at 25 °C and 95% relative humidity and plunged into liquid ethane using a Leica EM-GP Automatic Grid Plunger. The specimens were equilibrated at −178 °C in the microscope prior to the imaging process. Time-dependent cryo-TEM samples were prepared just before the plunging, using a stopwatch. The same assembly solution that was plunged at *t* = 1 min was used again at *t* = 5 min and *t* = 30 min in order to elucidate the structural evolution of the self-assembled crystals.

### Femtosecond transient absorption

Femtosecond transient absorption spectroscopy was performed by a methodology analogous as described previously [36], using a system based on a mode-locked Ti:sapphire oscillator (Spectra Physics MaiTai). The oscillator produces a train of <120 fs pulses (bandwidth ≈ 10 nm FWHM), with a peak wavelength centered at 800 nm. The weak oscillator pulses are amplified by a chirped pulse regenerative amplifier (Spectra Physics Spitfire ACE). The pulses are first stretched, then regeneratively amplified in a Ti:sapphire cavity, pumped by a pulsed Nd:YLF laser (Spectra Physics Empower 45) operating at 1 kHz. After the pulse has been amplified and recompressed, the energy is about 5.0 mJ in a train of 1 kHz pulses and about 1 mJ is used in the transient absorption setup. An independent pump pulse is obtained by pumping an optical parametric amplifier (Spectra Physics OPA-800CF) that produces 120 fs pulses tunable from 300 nm to 3 μm. The output power of the OPA is a few microjoule (depending on the chosen wavelength) at 1 kHz. The pump beam is mechanically chopped at half the amplifier repetition rate. The chopper (C-995 TTI) is synchronized with the Spitfire pulses. Normally, a few thousand pulse pairs (pump on/pump off) are averaged to produce a transient absorption spectrum with a noise level below 0.3 mOD. A small portion of the remaining amplified pulse is used to generate a white-light continuum as a probe pulse. To this end, the Ti:sapphire beam is focused onto a 3 mm thick sapphire disk by a 10 cm focal length lens, and the numerical aperture of the beam is controlled by an iris placed in front of the lens to obtain a stable and smooth white-light continuum. The resulting beam is passed through a Raman notch filter in order to remove the remains of the 800 nm fundamental beam from the probe white-light continuum. The pump and probe pulses are crossed in the sample at a small angle while maintaining a magic angle between the pump and probe polarizations. The remains of the pump pulse are removed by passing the probe through an iris, and it is then imaged onto an optical fiber that brings it into a fiber optic interface, which focuses the light onto the entrance slit of a Jobin Yivon Triax 180 spectrograph. The light is normally dispersed by a 300 gr/mm grating onto a fast CCD camera (Andor Newton DU-970N-UV, operating at 1,000 spectra per second using "crop mode"). The whole setup is controlled by the National Instruments LabView software. A variable neutral-density filter was employed to adjust the pump power for studying the power dependence. The pump power intensities were measured using an Ophir powermeter with a photodiode sensor in proximity to the sample. The excitation densities were calculated for a laser spot of 300 μm diameter on the sample. This diameter was measured by placing a beamprofiler (Ophir Beamstar FX33) at the sample position and determination the 4-sigma (95% of the power) parameter. In the reported experiments, the pump was tuned to 525 or to 590 nm, and the optical densities of the samples (in 4 mm and 2 mm optical path length cuvettes) were kept between 0.2 and 0.5 at the excitation wavelength. The instrument response function (300 fs) was recorded by repetition of the experiments, with the sample replaced by the pure solvent and keeping all other parameters unchanged. Spectral corrections and analysis were performed using the Surface Xplorer Pro (Ultrafast Systems) and Origin 7.5 (OriginLab) softwares.

## Supporting Information

File 1 General information, synthesis, molecular modeling, and further experimental details.

## References

[1] Brus L (1991). Appl Phys A: Solids Surf.

[2] Kelly K L, Coronado E, Zhao L L, Schatz G C (2003). J Phys Chem B.

[3] Alivisatos A P (1996). Science.

[4] Halperin W P (1986). Rev Mod Phys.

[5] Peng X, Schlamp M C, Kadavanich A V, Alivisatos A P (1997). J Am Chem Soc.

[6] Colvin V L, Schlamp M C, Alivisatos A P (1994). Nature.

[7] Kasai H, Nalwa H S, Oikawa H, Okada S, Matsuda H, Minami N, Kakuta A, Ono K, Mukoh A, Nakanishi H (1992). Jpn J Appl Phys, Part 1.

[8] Iida R, Kamatani H, Kasai H, Okada S, Oikawa H, Matsuda H, Kakuta A, Nakanishi H (1995). Mol Cryst Liq Cryst Sci Technol, Sect A.

[9] Fu H-B, Wang Y-Q, Yao J-N (2000). Chem Phys Lett.

[10] Kasai H, Kamatani H, Okada S, Oikawa H, Matsuda H, Nakanishi H (1996). Jpn J Appl Phys, Part 1.

[11] Fu H-B, Yao J-N (2001). J Am Chem Soc.

[12] Baba K, Kasai H, Masuhara A, Oikawa H, Nakanishi H (2009). Jpn J Appl Phys.

[13] Fery-Forgues S (2013). Nanoscale.

[14] Nakanishi H, Katagi H (1998). Supramol Sci.

[15] Rosenne S, Grinvald E, Shirman E, Neeman L, Dutta S, Bar-Elli O, Ben-Zvi R, Oksenberg E, Milko P, Kalchenko V (2015). Nano Lett.

[16] Schierl C, Niazov-Elkan A, Shimon L J W, Feldman Y, Rybtchinski B, Guldi D M (2018). Nanoscale.

[17] Kasai H, Murakami T, Ikuta Y, Koseki Y, Baba K, Oikawa H, Nakanishi H, Okada M, Shoji M, Ueda M (2012). Angew Chem, Int Ed.

[18] Zhao Y S, Fu H, Peng A, Ma Y, Xiao D, Yao J (2008). Adv Mater (Weinheim, Ger).

[19] Komai Y, Kasai H, Hirakoso H, Hakuta Y, Okada S, Oikawa H, Adschiri T, Inomata H, Arai K, Nakanishi H (1998). Mol Cryst Liq Cryst Sci Technol, Sect A.

[20] Jiang H, Zhang K K, Ye J, Wei F, Hu P, Guo J, Liang C, Chen X, Zhao Y, McNeil L E (2013). Small.

[21] Hiremath R, Basile J A, Varney S W, Swift J A (2005). J Am Chem Soc.

[22] Kang J F, Zaccaro J, Ulman A, Myerson A (2000). Langmuir.

[23] Briseno A L, Aizenberg J, Han Y-J, Penkala R A, Moon H, Lovinger A J, Kloc C, Bao Z (2005). J Am Chem Soc.

[24] Kim B J, Yu H, Oh J H, Kang M S, Cho J H (2013). J Phys Chem C.

[25] Balakrishnan K, Datar A, Oitker R, Chen H, Zuo J, Zang L (2005). J Am Chem Soc.

[26] Balakrishnan K, Datar A, Naddo T, Huang J, Oitker R, Yen M, Zhao J, Zang L (2006). J Am Chem Soc.

[27] Zhang Z, Zhang X, Zhan C, Lu Z, Ding X, He S, Yao J (2013). Soft Matter.

[28] Che Y, Datar A, Balakrishnan K, Zang L (2007). J Am Chem Soc.

[29] Weissbuch I, Lahav M, Leiserowitz L (2003). Cryst Growth Des.

[30] Vekilov P G (2010). Cryst Growth Des.

[31] Jehannin M, Rao A, Cölfen H (2019). J Am Chem Soc.

[32] Sear R P (2012). Int Mater Rev.

[33] Davey R J, Schroeder S L M, ter Horst J H (2013). Angew Chem, Int Ed.

[34] Tsarfati Y, Rosenne S, Weissman H, Shimon L J W, Gur D, Palmer B A, Rybtchinski B (2018). ACS Cent Sci.

[35] Shahar C, Dutta S, Weissman H, Shimon L J W, Ott H, Rybtchinski B (2016). Angew Chem, Int Ed.

[36] Shahar C, Baram J, Tidhar Y, Weissman H, Cohen S R, Pinkas I, Rybtchinski B (2013). ACS Nano.

[37] Krieg E, Rybtchinski B (2011). Chem – Eur J.

[38] Krieg E, Niazov-Elkan A, Cohen E, Tsarfati Y, Rybtchinski B (2019). Acc Chem Res.

[39] Matern J, Dorca Y, Sánchez L, Fernández G (2019). Angew Chem, Int Ed.

[40] Matsumoto N M, Lafleur R P M, Lou X, Shih K-C, Wijnands S P W, Guibert C, van Rosendaal J W A M, Voets I K, Palmans A R A, Lin Y (2018). J Am Chem Soc.

[41] Basak D, Ghosh S (2013). ACS Macro Lett.

[42] Yin M, Shen J, Pisula W, Liang M, Zhi L, Müllen K (2009). J Am Chem Soc.

[43] Cormier R A, Gregg B A (1998). Chem Mater.

[44] Kazmaier P M, Hoffmann R (1994). J Am Chem Soc.

[45] Klebe G, Graser F, Hädicke E, Berndt J (1989). Acta Crystallogr, Sect B: Struct Sci.

[46] Zang L, Che Y, Moore J S (2008). Acc Chem Res.

[47] Würthner F, Bauer C, Stepanenko V, Yagai S (2008). Adv Mater (Weinheim, Ger).

[48] Glatter O, Kratky O (1982). Small angle x-ray scattering.

[49] Tidhar Y, Weissman H, Wolf S G, Gulino A, Rybtchinski B (2011). Chem – Eur J.

[50] Wasielewski M R (2006). J Org Chem.

[51] Pope C E, Swenberg M (1999). Electronic Processes in Organic Crystals and Polymers.

[52] Engel E, Leo K, Hoffmann M (2006). Chem Phys.

[53] Ahrens M J, Sinks L E, Rybtchinski B, Liu W, Jones B A, Giaimo J M, Gusev A V, Goshe A J, Tiede D M, Wasielewski M R (2004). J Am Chem Soc.

[54] Suna A (1970). Phys Rev B.

[55] Inoue A, Yoshihara K, Nagakura S (1972). Bull Chem Soc Jpn.

[56] Marciniak H, Li X-Q, Würthner F, Lochbrunner S (2011). J Phys Chem A.

[57] Rehhagen C, Stolte M, Herbst S, Hecht M, Lochbrunner S, Würthner F, Fennel F (2020). J Phys Chem Lett.

[58] Bisri S Z, Takenobu T, Yomogida Y, Shimotani H, Yamao T, Hotta S, Iwasa Y (2009). Adv Funct Mater.

