# Supplementary material for: Control over size, shape, and photonics of self-assembled organic nanocrystals
Source: Beilstein J Org Chem. 2021 Jan 6;17:42–51. doi: 10.3762/bjoc.17.5 (PMC7801800; doi:10.3762/bjoc.17.5)
Supplement: File 1 — General information, synthesis, molecular modeling, and further experimental details. [file Beilstein_J_Org_Chem-17-42-s001.pdf]

BEILSTEIN JOURNAL OF ORGANIC CHEMISTRY

Supporting Information

for

Control over size, shape, and photonics of self-assembled organic nanocrystals

Chen Shahar, Yaron Tidhar, Yunmin Jung, Haim Weissman, Sidney R. Cohen, Ronit Bitton, Iddo Pinkas, Gilad Haran and Boris Rybtchinski

Beilstein J. Org. Chem. **2021,** *17,* 42–51. doi:10.3762/bjoc.17.5

General information, synthesis, molecular modeling, and further experimental details

License and Terms: This is a supporting information file under the terms of the Creative Commons Attribution License (https://creativecommons.org/licenses/by/4.0). Please note that the reuse, redistribution and reproduction in particular requires that the author(s) and source are credited and that individual graphics may be subject to special legal provisions.
The license is subject to the *Beilstein Journal of Organic Chemistry* terms and conditions: (https://www.beilstein-journals.org/bjoc/terms)

General information

The ^{1}H and ^{13}C NMR spectra were recorded at 20 °C on a 300 MHz NMR spectrometer (Bruker). ^{1}H and $^{13}C\{^{1}H\}$ NMR chemical shifts are reported in parts per million (ppm) downfield from tetramethylsilane (δ scale). ^{1}H NMR chemical shifts were referenced to the ^{1}H signal of the residual $CHCl_3$ in $CDCl_3$ (7.26 ppm). In $^{13}C\{^{1}H\}$ NMR measurements, the signal of $CDCl_3$ (77.16 ppm) was used as a reference. Coupling constants (*J*) are reported in Hertz (Hz), and splitting patterns are designated as s (singlet), d (doublet), t (triplet), q (quartet), m (multiplet), b (broad). Electrospray ionization (ESI) mass spectrometry was performed using a Micromass Platform instrument. UV–vis absorption and fluorescence measurements were carried out on a Cary-5000 spectrometer (Varian) and a Cary Eclipse fluorimeter (Varian), respectively. Quantum yields were determined following the standard procedure[1] using a sulforhodamine 101 solution in ethanol (λ_{abs} = 576 nm, λ_{em} = 592 nm, φ = 0.9) as a reference.

Atomic force microscopy (AFM) measurements were made by a methodology analogous as described in [36] (see references in the Main Manuscript), using an NTEGRA AFM equipped with a SF005 head and 10×10 μm^2 scanner (NT-MDT, Zelenograd). Scans were made in tapping/semicontact mode using an AC240 TS silicon probe (Olympus).

The heights of the belt-like structures were determined using individual cross-sections and histogram height analysis. For the latter, the most probable pixel heights over a region containing primarily background with a minimal amount of debris were subtracted from average heights of regions encompassing a single layer of the assembly films. Sample preparation: AFM measurements were carried out on a (100) Si wafer substrate with 200 nm oxide. The different samples were prepared by incubating 10 µl of a 1×10^{-4} M solution on a 1×1 cm^2 Si substrate for 1 minute before blotting the excess liquid in order to avoid the formation of stacked deposits of the assemble material upon drying.

Small-angle X-ray scattering (SAXS): Small-angle X-ray scattering measurements were performed using the SAXSLAB GANESHA 300-XL_system with Cu

Kα radiation generated by a sealed microfocused tube (Genix 3D Cu source with integrated monochromator) powered at 50 kV and 0.6 mA and three pinholes collimation. The scattering patterns were recorded by the Pilatus 300 K detector.
The scattering intensity $I(q)$ was recorded in the interval $0.012 < q < 0.7$ Å^{-1}, where q is defined as $q = \frac{4\pi}{\lambda}\sin\theta$ where 2θ is the scattering angle, and λ is the radiation wavelength (1.542 Å). The solution under study was sealed in a thin-walled capillary (glass) of about 1.5 mm diameter and a 0.01 mm wall thickness. Measurements were performed under vacuum at ambient temperature. The 2D SAXS images were azimuthally averaged to produce one-dimensional profiles of the intensity I vs q using the two-dimensional data reduction program SAXSGUI. The scattering spectra of the capillary and solvent were also collected and subtracted from the corresponding solution data using the Irena package for analysis of small-angle scattering data[1]. No attempt was made to convert the data to an absolute scale. The data analysis was based on fitting the scattering curve to an appropriate model by a least-squares method using the software provided by NIST[2] (NIST SANS analysis version 7.0 on IGOR).

Superresolution microscopy imaging was performed using a custom-built stochastic optical reconstruction microscopy (STORM) [3-5] system equipped with an objective for total internal reflection (UAPON 100×OTIRF, N.A. 1.49, Olympus). Additional lenses were added to achieve a final magnification of 240× on an EMCCD camera (iXon^{EM} +897 back-illuminated, Andor). Sample preparation: A drop of the sample was allowed to interact for few seconds on a freshly cleaned cover glass by using 1 N NaOH and then, unbound material was washed extensively with distilled water. The measurement was done after dropcasting a drop of water on the sample. The regions of interest were screened and selected under weak illumination with a 532 nm laser (Cobolt), and STORM measurements were then performed using a ≈30 mW (corresponding to ≈18.7 kW/cm^2) illumination power. 12000 frames were recorded, divided into 5 series of movies at frame rates of 33 Hz and applying the frame transfer feature of the EMCCD camera. In each frame, individual emitters were found and localized using a standard 2D Gaussian fitting procedure [6]. The sample drift was corrected movie by movie based on a selected fiducial point that appeared constantly

in all series of the movies.

Synthesis

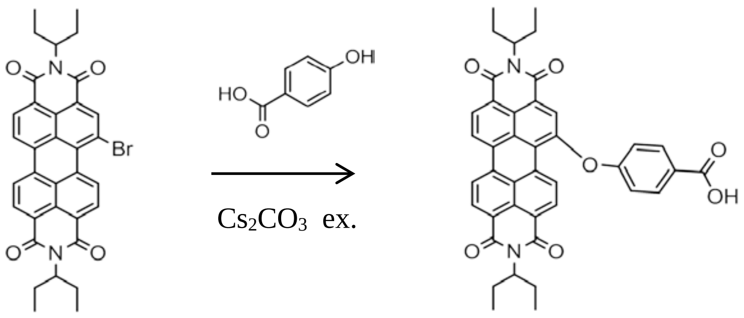

1

The synthesis was performed according to [7]. The characterization of **1** is in accordance with published data [7]:

^{1}**H NMR** ($CDCl_3$): 9.39 (d, 1H, $^{3}J_{HH}$= 9 Hz, perylene-H), 8.71 (m, 4H, overlapped perylene-H), 8.61 (d, 1H, $^{3}J_{HH}$= 6 Hz, perylene-H), 8.31 (s, 1H, perylene-H), 8.18 (d, 2H, $^{3}J_{HH}$= 9 Hz, phenyl-H), 7.21 (d, 2H, $^{3}J_{HH}$= 9 Hz, phenyl-H), 5.05 (m, 2H, $N(C\mathit{H}(CH_2CH_3)_2)$), 2.22 (m, 4H, $N(CH(C\mathit{H}_2CH_3)_2)$), 1.93 (m, 4H, $N(CH(C\mathit{H}_2CH_3)_2)$), 0.91 (t, 12H, $^{3}J_{HH}$= 7.5, $N(CH(CH_2C\mathit{H}_3)_2)$). ^{13}C NMR ($CDCl_3$): 178.32, 172.33, 168.64, 165.89, 159.51, 135.15, 133.07, 128.55, 126.61, 123.77, 122.72, 118.44, 110.64, 99.97 57.9, 57.65, 29.65, 24.96, 11.31. MS-ESI (m/z): calculated for $[M-H]^-$ $C_{41}H_{33}N_2O_7$ 665.23; found: 665.17. UV/vis ($CHCl_3$): λ_{max}/nm (ε/M^{-1} cm^{-1}) = 456 (3440), 488 (9070), 522 (13950). Fluorescence ($CHCl_3$): λ_{max} = 524 nm; quantum yield Φ_f=0.47.

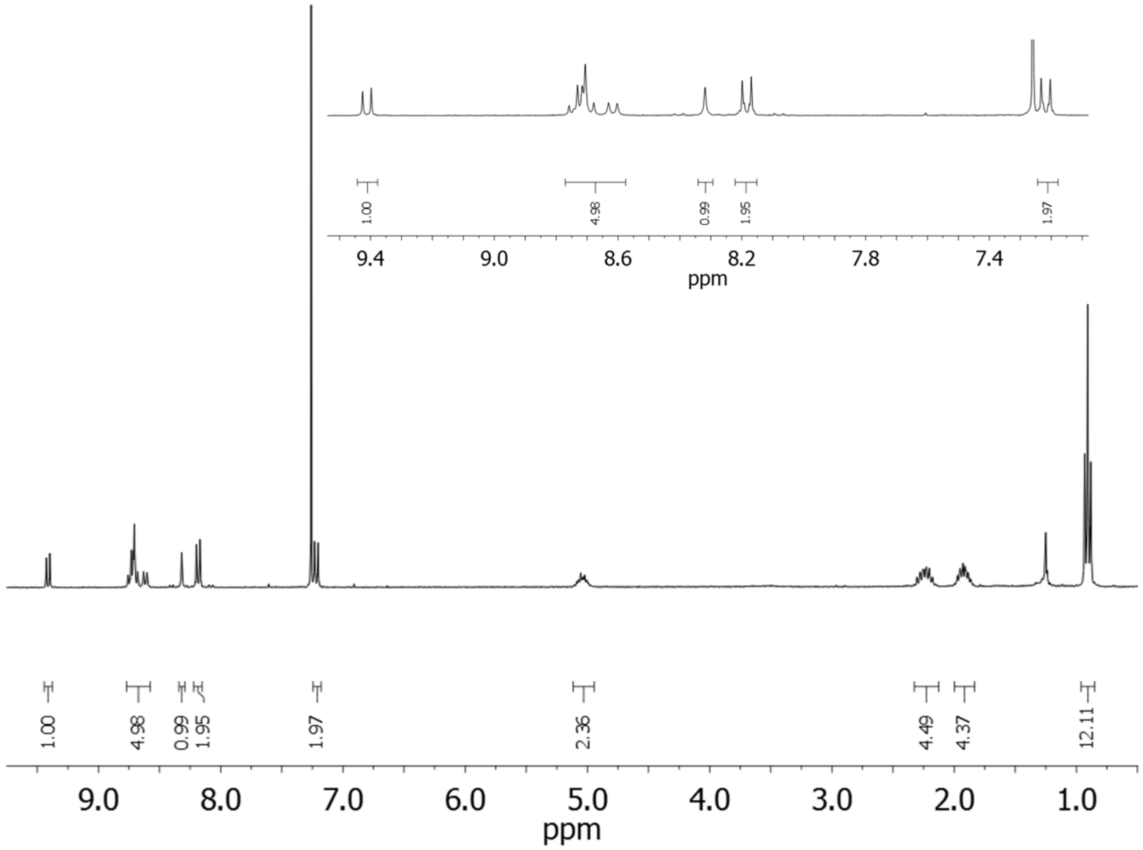

Figure S1: ^{1}H NMR spectrum of **1** in $CDCl_3$

Molecular modeling

Molecular modeling was performed using SCIGRESS version 2.2.0, build 3624 (Fujitsu). Molecular mechanics force fiel (augmented MM3) was used for geometry optimization. It is important to note that the hydrophobic interactions involving an explicit water network cannot be satisfactorily modeled for such large systems. Our models were constructed to fit the structures observed in cryo-TEM images, with geometric dimensions and difference in the contrast serving as modeling guidelines. The models of the assembled compound **1** represent the best fit to the cryo-TEM, AFM, and SAXS data.

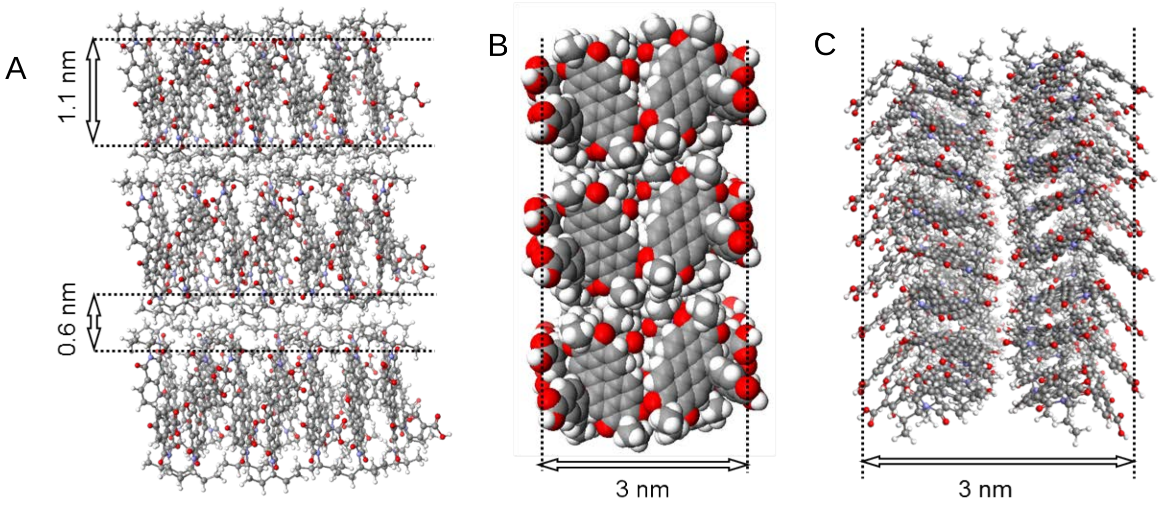

Figure S2: Molecular model of crystalline assemblies. A) Side view presenting the interacting stacks and their dimensions corresponding to the stripy fine-structure observed in cryo-TEM and TEM. B) and C) Cross-section and top view, respectively, demonstrating the bilayer structure composed of two adjacent PDI cores. The measured distance (3 nm) corresponds to the oxygen–oxygen distance between the carboxylic groups of two PDIs.

AFM

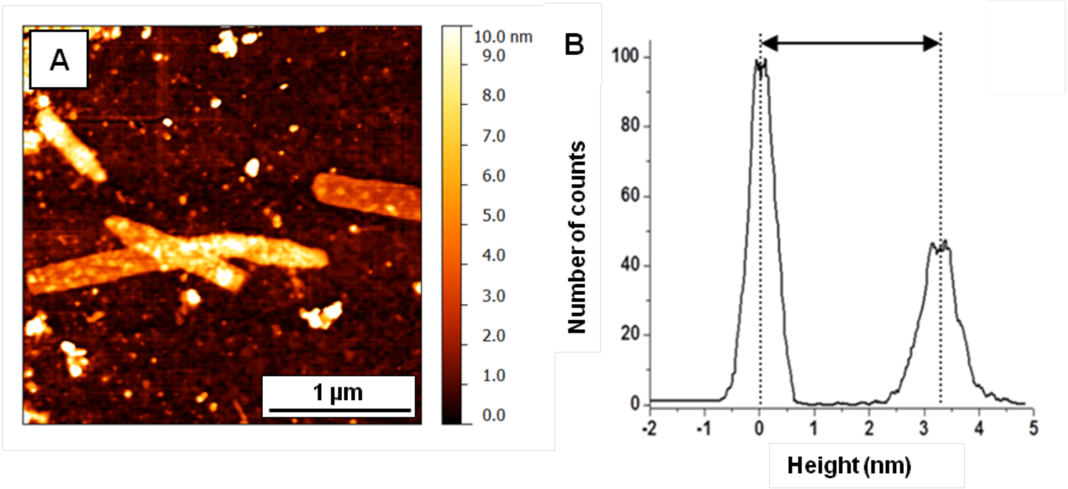

Figure S3: AFM measurements and histogram-height analysis of assembled **1** (5% THF system). A) AFM image. B) Histogram representing the height difference distribution of the air-dried assemblies on a Si surface; the height difference between the substrate and the crystalline material is indicated by an arrow and equals 3.2 ± 0.4 nm, in agreement with the oxygen–oxygen distance between the carboxylic acid groups of two PDIs obtained by molecular modeling (3 nm).

SAXS

Modeling of small-angle scattering patterns: The scattering intensity of a monodispersed system of particles of identical shape can be described by the following equation [8]:

(S1) $$I(q) = NP(q)S(q)$$

Where N is the number of particles per unit volume, $P(q)$ is the form factor revealing the specific size and shape of the scatters and $S(q)$ is the structure factor that accounts for the interparticle interactions. In dilute solutions, where the interactions between the objects can be neglected, $S(q)$ is equivalent to 1. In a polydispersed system of particles having identical shapes, the total intensity scattered can be described by the following equation:

(S2) $$I(q) = N\int_0^\infty D_n(R)P(q,R)dR$$

where $D_n(R)$ is a distribution function and $D_n(R)dR$ is the number of particles, the size of which is between R and R + dR, per unit volume of sample.

The scattered intensity for randomly distributed lamellae of a uniform scattering length density and polydispersed thickness is given by the following equation:

(S3) $$I(q) = \frac{2\pi P(q)}{\delta q^2}$$

Where the form factor is:

(S4) $$P(q) = \frac{2\Delta\rho^2}{q^2}\left[1-\cos(q\delta)e^{-q^2\sigma^2/2}\right]$$

$\Delta\rho$ is the scattering length density difference between the lamellae and solvent, δ is the bilayer thickness and σ is the variation in bilayer thickness. No interlamellar structure factor is calculated in this model.

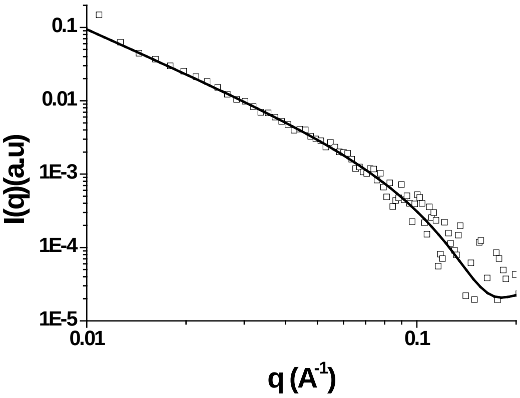

Figure S4: SAXS curve of 6×10^{-4} M compound **1** in 5% THF aqueous solution. The solid line represents the best fit to a model of dilute polydispersed lamellae.

It should be noted that the SAXS pattern of the assembled solution does not exhibit characteristics of a crystalline structure (i.e., there is no indication of Bragg peaks) due to low contrast of the diluted lamellar structure. The same sample was remeasured a few weeks later, when some sedimentation started to take place in the capillary. The resulting scattering demonstrated a distinct peak at q = 0.351 which corresponds to d-spacing of ≈1.7 nm. The obtained value is in good agreement with the molecular modeling and the FFT spacing obtained from the TEM and cryo-TEM images (1.6 nm).

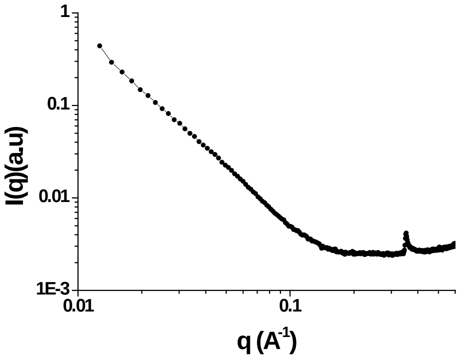

Figure S5: SAXS curve of the precipitant of compound **1** in 5% THF aqueous solution.

Pathway dependence, control experiments

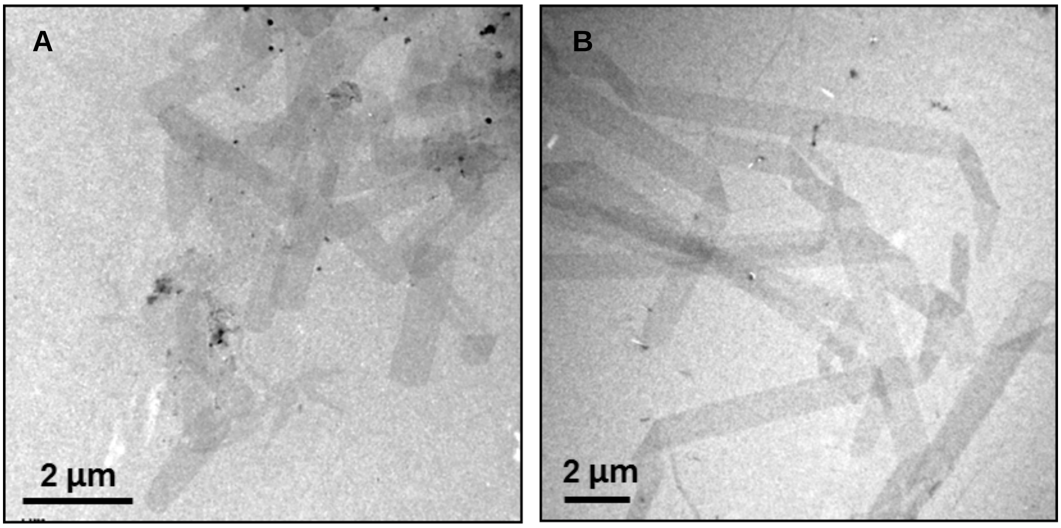

Figure S6: A) Preaged 5% THF adjusted to 10% THF. B) Preaged 5%→0% THF adjusted to 5% THF, as described in the text.

Time-dependent cryo-TEM experiments

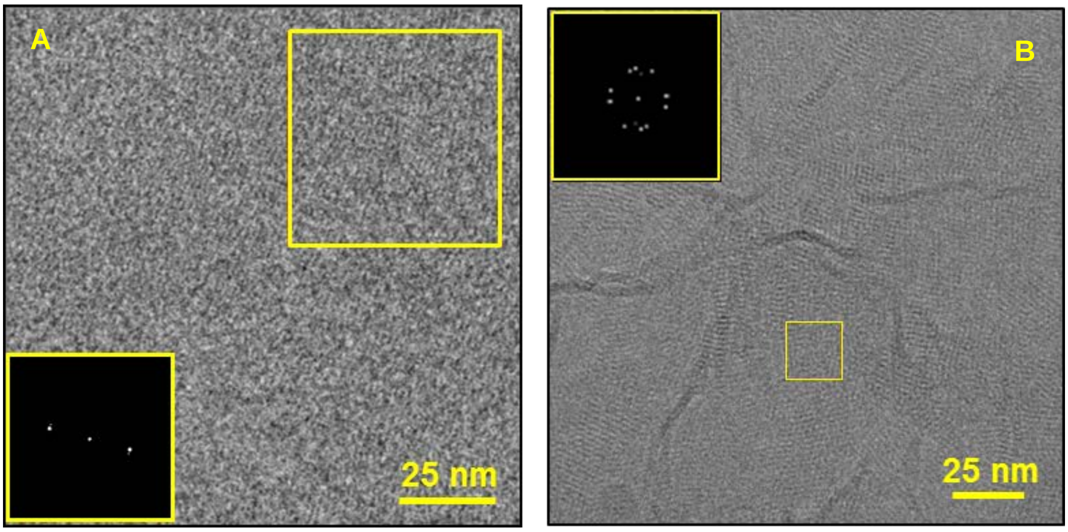

Figure S7: Cryo-TEM images of 1×10^{-4} M solution of **1** in THF/water after 5 min aging time. A) 5% THF and B) 10% THF.

pH effect

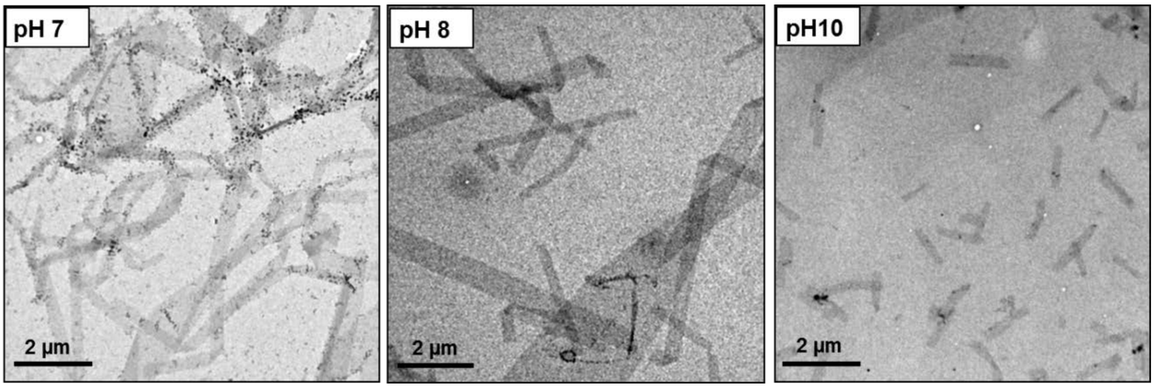

Figure S8: TEM images of 1×10^{-4} M compound **1** assembled in 5% THF at different pH conditions. At lower pH the stabilization of the amphiphilic building block is compromised, and smaller disordered aggregates are observed together with crystalline structures.

Transient absorption, power dependence

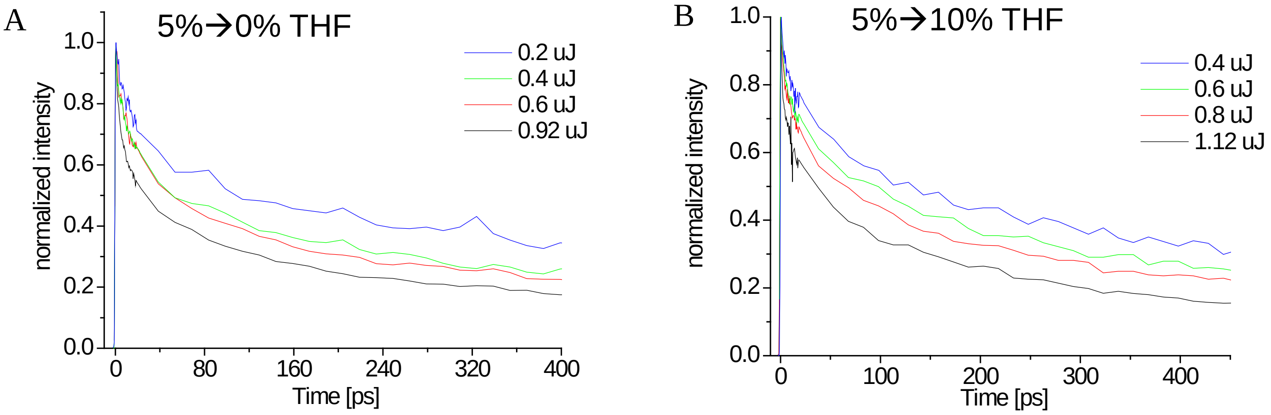

Figure S9: Transient kinetic traces taken at different laser powers (probed at 755 nm). A) in 5→0% THF solution; B) 5→10% THF, as described in the text.

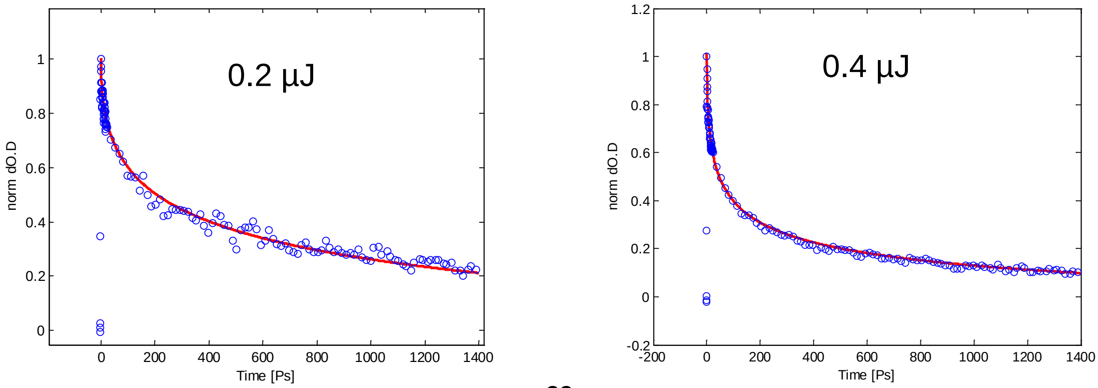

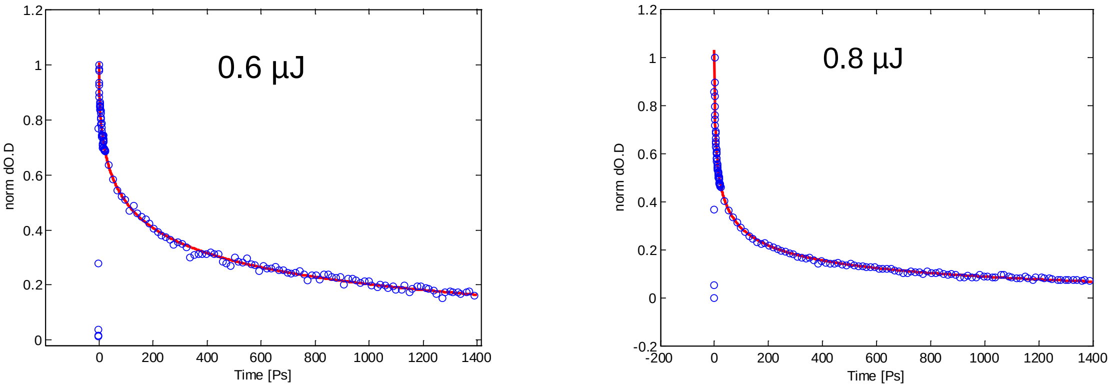

Figure S10: Fitted kinetic traces of 5% THF assembly of compound **1** at different laser powers.

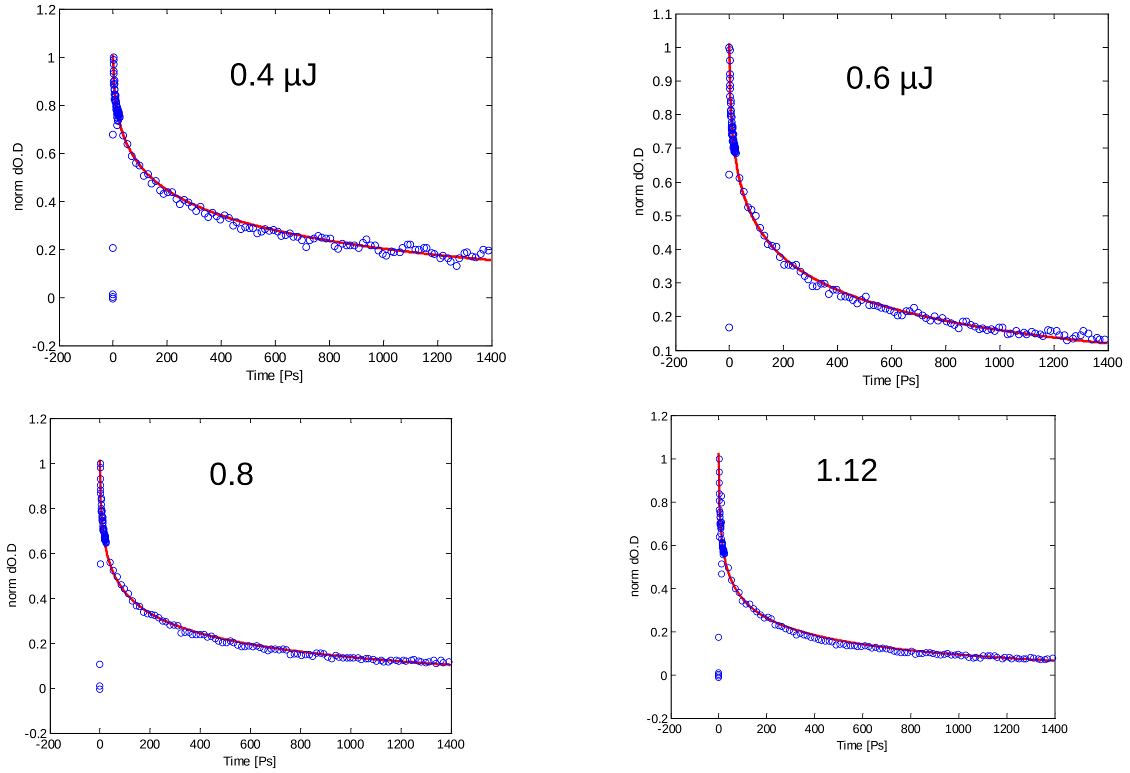

Figure S11: Fitted kinetic traces of the control experiment (5%→10% THF assembly of compound **1** at different laser powers.

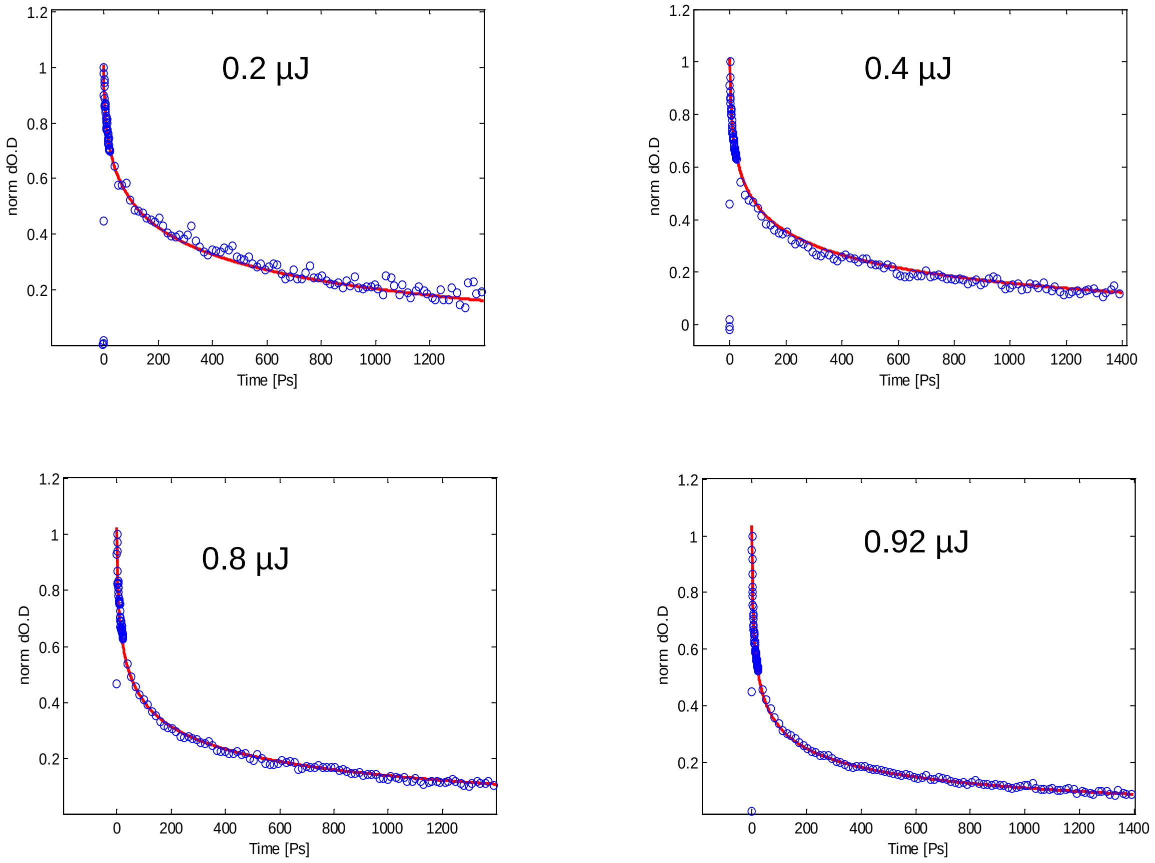

Figure S12: Fitted kinetic traces of the control experiment, 0% THF at different laser powers.

Superresolution microscopy

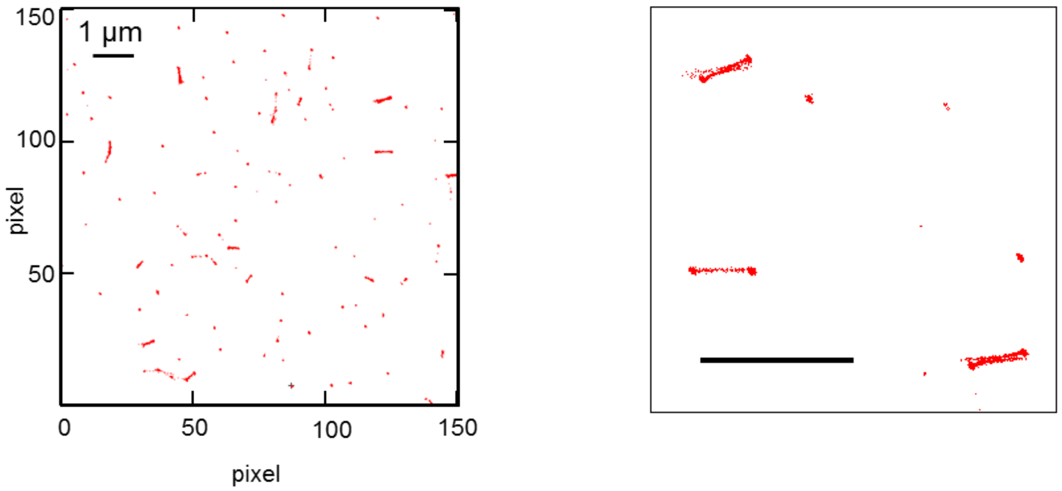

Figure S13: Superresolution microscopy images of the 5% THF system. The left panel shows the superresolution image of an area covered with the 5% THF structures. Essentially all

emitting molecules were localized on lines likely to be the edges of crystals, with a strong preference for emission at the corners. The right panel shows a detail from the same image (scale bar = 1μm).

[1] Ilavsky, J.; Jemian, P. R. *J. Appl. Crystallogr.* **2009**, *42*, 347–353.

[2] Kline, S. R. *J. Appl. Crystallogr.* **2006**, *39*, 895–900.

[3] Rust, M. J.; Bates, M.; Zhuang, X. *Nat. Methods* **2006**, *3*, 793–795.

[4] Zhuang, X. *Nat. Photonics* **2009**, *3*, 365–367.

[5] Patterson, G.; Davidson, M.; Manley, S.; Lippincott-Schwartz, J. *Annu. Rev. Phys. Chem.* **2010**, *61*, 345–367.

[6] Bates, M.; Jones, S. A.; Zhuang, X. *Cold Spring Harb. Protoc.* **2013**, *2013*, 498–520.

[7] Shahar, C.; Dutta, S.; Weissman, H.; Shimon, L. J. W.; Ott, H.; Rybtchinski, B. *Angew. Chem. Int. Edit.* **2016**, *55*, 179-182.

[8] Glatter O.; Kratky O. *Small Angle X-ray Scattering*; Academic Press: London, 1982.
